# Horse-breeding1Read before the Montreal Veterinary Medical Association.

**Published:** 1896-07

**Authors:** J. A. Ness

**Affiliations:** Howick (Quebec), Canada


### HORSE-BREEDING.[1]

By J. A. NESS, D.V.S.,
HOWICK (QUEBEC), CANADA.

THE subject which I have chosen to bring before your notice this evening, viz., horse-breeding, may be somewhat of a digression from the usual essays presented to the Society this session, but one of no little importance to the practitioner of comparative medicine and veterinary science, and a knowledge of which will aid him materially in practice. The breeding of domesti-

[1] Read before the Montreal Veterinary Medical Association.

cated animals for the various purposes to which they are adapted has been practised from the earliest times. The oldest writers on agriculture gave directions for the breeding and improvement of live-stock, and many of their maxims are repeated to-day as the best practical guides to the breeder. Before going into detail on any one breed we will consider some of the principles which underlie successful breeding and must be thoroughly known. First and most important of all is heredity. Heredity, so well expounded by Darwin, is the perpetuation of like characteristics in parent and offspring. The general principles which govern the transmission of hereditary qualities from parent to offspring are without doubt the same throughout all animal life. This characteristic has long been known, and is the result of observation forcibly expressed in the familiar aphorism, "like produces like," and, as an inference of this generally accepted law of the animal organization, we would naturally breed from the best. This selection as "best" must not be continually changing, else no progress will be made, as heredity only transmits with certainty what has become a fixed characteristic in the breed.

Oliver Wendell Holmes, in his *Autocrat of the Breakfast Table*, gives utterance to his faith and belief of heredity when he says, "Of the human family, I go always, other things being equal, for the man who inherits family traditions and the cumulative humanities of at least four or five generations." It is one of the principles of heredity that when there is great uniformity in a species divergences from the usual type in the offspring are rare; but when this uniformity, no matter from what cause, has been broken up, divergences are frequent and great, although there is always present a tendency, more or less powerful, to revert to the original type. This tendency is most frequently manifested when breeds or races, widely differing in their present forms, are crossed upon each other. In such cases it frequently happens that the progeny resembles neither parent, but shows strong marks of the type from which it has originally, or rather both of its ancestors originally, sprung.

This tendency to reversion in different breeds of domestic animals when crossed accounts for many of the disappointments which breeders experience in their efforts to improve their stock, and serves greatly to complicate the breeding problem. Bakewell, who was one of the first improvers of the live-stock of Great Britain, regarded the animals on his farm as wax in his hands, out of which in good time he could mould any form that he desired to create. He believed that the familiar maxim, "like begets like," was not limited to a general similarity of offspring to parent, but extended to the minutest details of the animal organism.

What can be said on heredity with regard to normal characters also applies to disease. Any abnormal peculiarities of the animal organization constituting disease, whether of structure or function, are liable to be transmitted from parent to progeny. Heredity disease may be congenital—*i. e.*, make its appearance at the time of birth, or a considerable length of time may elapse before any indications of its presence are observed, in which case a predisposition or tendency to the disease is inherited, which often requires some external exciting cause for its development. There are certain diseases that are transmitted with greater uniformity than others, yet a predisposition to almost every known form of disease is likely to become hereditary even if the influence which determines its transmission is not sufficiently intense to render it congenital. When the general constitutional predisposition is inherited the conditions to which the animal is subjected, as to food, exposure, etc., may have an influence in determining the particular organ in which the disease is developed. Without a further enumeration of details it may be said that every peculiarity of the animal organization is influenced by heredity. Sometimes we may meet with striking differences between the parent and progeny, but the points of resemblance are greater than those of difference, as a rule. It may seem as though I have dwelt too long on the subject of heredity, but it cannot be too forcibly impressed on the minds of would-be successful breeders.

In the selection of breeding-stock the first step for the breeder is to decide what sort of a horse will bring him the best returns. Success in the breeding of live-stock must be measured by the actual value of the products and the profits that may be derived therefrom. If his interest leads him to breed for the race-course he must keep constantly in mind the fact that for this purpose, whether for running or trotting, speed and endurance of the very highest order are indispensable; but if the breeding necessitates a direct livelihood therefrom, he had better leave the breeding of race-horses to the man with the money. For the ordinary breeder must consider the horse that sells best on the market, or rather the horse for which there is the greatest demand, and that is in order of merit: First, the large, stylish, high-stepping carriage or coach horse. With the proper stock to breed from such horses may be bred with a good degree of certainty. Such as prove deficient in style and action make good express horses, but to command the best prices they must be rather "rangy" and possess higher knee-action than is desirable for ordinary work. Second, the heavy draught-horse, from fifteen hundred pounds upward, as heavy as possible, without departing from quality to obtain such weight. Further remarks will be confined to the draught-horse in particular, as I have had more experience with this class.

In the selection of the sire you must first investigate his pedigree, which, as you well know, consists of the names of the ancestors for a greater or less number of generations. Its value consists not so much in the number of generations through which the ancestry can be traced to some progenitor, as in the quality or character of the ancestry or the merits of individuals that compose it.

Having satisfied yourself of his breeding, you now secure the form of horse desired. There are, no doubt, several breeds of draught-horses, all of which have their qualities and faults; but for the purpose of description I shall try and enumerate the points necessary in good draught-horses without taking up any special breed, which are, beginning at the head, as follows: The head should be of medium size, in proportion to that of the body; the forehead broad between the eyes, tapering upward in the direction of the ears; the jaw broad but not large or loaded with flesh; the nasal bones, if not straight, slightly arched, but not dish-faced; the muscles not too refined or tapering, with the nostrils wide; the eye bright and dark, full and vigorous, yet mild; the ears of good size, tapering to a point, neither hanging, showing sluggishness, not prick-eared, but with frequent motion indicative of a good disposition. Such a description of the head rarely finds a vicious temperament, which latter must be detested in a breeding sire.

The neck should be of good length, clean-cut at the jaws, well set on at the shoulder, with gradual tapering, but slight arch from the occiput to the highest point of the withers. The chest must be broad, full, and deep—a hollow chest suggests decided weakness. The shoulder should be closely knit at the withers and oblique, though not necessarily so much so as in the race-horse. The humerus should form a very obtuse angle with the scapula, or else the animal cannot well advance the limb in motion. He should be well up at the withers. The back should be short and strong; the ribs of great depth and rounded, barrel-shaped; he should be full at the heart, and girth well, allowing plenty of room for the expansion of healthy lungs. The length, or rather depth of body at the last rib, should be great, as this is too often a defect in the draught-horse, and a serious one, as it suggests a poor feeder and also gives him a leggy appearance. The hind-quarters of good length, well-muscled and round, the thigh being well developed and strong.

The last in consideration, but by no means the least, are legs and feet. As our dean forcibly expresses himself on this point, "No foot, no horse," and observation should confirm the statement. The indications of a good leg are firmness, hardness, and smoothness to the touch, showing an entire absence of adipose tissue; large, well-defined joints entirely free from any abnormal appendages. The knee-joint should be broad and flat. The hock wide from before backward, giving the leg a somewhat crooked appearance rather than straight, the former giving strength. The length of bone short from the knee and hock to the pastern. When standing the hocks should tend to be close together. The large metacarpals should present a flat side-view, rounded anteriorly and tapering posteriorly, and of good size below the knee; the tendons at this point well defined and strong. The pasterns of medium length and set on rather oblique so as to give the necessary elasticity to counteract the concussion caused by the firm step. If long they are weak, if short and upright they are liable to knuckling.

The feet are, of course, very important. In shape the medium between a flat foot and a mule foot is preferable, of good size, with a large degree of concavity in the bottom. They must also be firm and elastic, without cracks and free from brittleness. The fore- and hindlegs must be set on straight, as an out- or an in-toed horse is decidedly objectionable.

All the preceding points are to be observed in the standing posture, and, when fully satisfied in these particulars, it is very essential to see that, having four good legs, the horse has the ability to use them properly; that he steps with a firm elastic tread; and, especially in the draught-horse, he must be a good smart walker; that the legs and feet do not get in the way of each other when he is in motion, but move freely without interference.

The general management of the sire is of great importance. It is a frequent mistake to have the sire in fine show condition when the season opens, and apparently in the pink of condition; but he is not nearly so well fitted for service in the stud as he would have been had this extra feeding been dispensed with. Such treatment is a drawback to the horse as a sure foal-getter. The point to be aimed at in the stable management of the sire is to feed, groom, and exercise so as to keep the horse up to the highest pitch of strength and vigor. He should be well and regularly fed on healthful, nutritious food, with plenty of exercise every day; let him be well groomed, the skin be kept clean by frequent brushing and rubbing, which also keeps the circulation in good order. Anything that adds to the health and strength and vigor of the horse will increase his sexual power; on the contrary, whatever tends to impair the general system will have a deleterious effect on the sexual organs. A healthy animal needs nothing but good food, pure air, plenty of exercise, with due attention to cleanliness and regularity of feeding and watering; and if such be attended to, but little will befall him unless by accident. He should be kept in a loose box at least twelve by sixteen feet, more if possible, which admits plenty of sunlight and is well ventilated; containing internally as few projections as possible upon which he may injure himself. It is well to have the loose box lined around with heavy material, standing out at the floor about one foot from the wall, sloping upward and inward to the wall, about three feet and a half high. By so doing it will be almost impossible for him to injure himself by rubbing or in any other way. In such a box he need not be haltered, and the owner may feel assured that the liability to injury is reduced to the minimum.

In the selection of the dam the same may be said as in the case of the sire. It is desirable that she should have a great depth of body with well-sprung ribs. As a rule, the female of any breed is somewhat finer in build than the male, and seldom reaches the relative weight of the sire. She must, as in the sire, be sound and free from any vicious temperament. What was said of overfeeding in the sire also applies to the dam, and is supposed to be the cause of barrenness to a certain extent in the dam. It is not advisable for her to breed before the age of three years. If she be younger or if she be too old, the foal may be small and weakly. The most fertile period in the life of the dam is usually from five to fifteen years of age. Above this age they usually become more uncertain, and regular breeders well up in years are comparatively rare; although there are exceptions to this, and instances are related of mares over twenty years of age producing healthy living foals, but such cases are rare.

When the time of foaling approaches the dam should be turned loose in a large box-stall, or, if the weather be mild, in a paddock. About the best time for foaling is the latter end of May, as there is an abundance of green food, and this is before the heat of summer sets in. The mare should be closely watched, as there are certain signs of parturition which rarely fail. The udder becomes greatly distended some time before foaling, but the teats seldom fill out full to the end until a day or two before the foal is dropped. When the mare is a valuable and well-bred one, and the foal expected to be a good one, it is well worth watching her closely, as many valuable animals have been lost which by a little attention at the right moment might have been saved. Other signs of parturition are wax-formation on the points of the teats, and a relaxation of the hindquarters, and a few hours before foaling she shows signs of uneasiness.

For a certain time previous to parturition she should be fed on soft food so as to keep her bowels open and free from constipation. Many mares are at best poor nurses, but her food may be made to greatly influence her yield of milk. The first milk after parturition, or colostrum, contains principles adapted to remove the meconium; on that account it is highly necessary that the newly-born foal should be supplied with the milk from its own dam, at least until the meconium has been expelled and the bowels have assumed their natural function. Until the approach of the time for weaning a foal should be permitted to have free access to its dam, otherwise in the young foal it may result in constipation or some other derangement of the digestive tract, which is serious in a very young foal. After eight or ten days of age the foal, if in good healthy condition, will probably give you but little trouble; but if it should necessitate treatment during the first four or five days of its life, little can be done, as the only internal treatment is by the dam, and that is unsatisfactory.

The weaning of the foal should be done gradually. It is always well to have the foal taught to eat all sorts of food before removing it from the dam, and it should be handled well and carefully from its birth. If such be carried out properly when weaning-time has come, which should be done at five or six months, confine the foal in a loose box, in which there is nothing for it to get entangled amongst, and feed on soft feed which you have already taught it to eat. It is much better to separate the foal and dam entirely, because if they are brought together it only lengthens the period of weaning. In the meantime the mother's diet should consist of dry feed and it is well to put her to work. The milk must be removed, but not milked dry every time. With the dry feed and work the secretion of milk will soon cease. From this time until the foal becomes a yearling it should be well fed, as a stunted foal never matures properly, and more colts are injured during the first six months after weaning by a too scanty supply of food than from the opposite extreme.

As soon as the foal is properly weaned it should have the run of a good pasture, as there is no food better than grass, no medicine so good as exercise, fresh air, and sunlight, and no exercise so profitable to young animals as that which may be taken just when they feel like it.

In conclusion, permit me to say that the subject of breeding is a wide and important one. There are omissions and points not yet touched upon, but which cannot be without making any one paper long in the extreme. Suffice it to say, that the general requirements of the successful breeding and raising of horses are plenty of good food, fresh air, and exercise for the development of the animal organism.

